# Small RNA NGS Revealed the Presence of Cherry Virus A and Little Cherry Virus 1 on Apricots in Hungary

**DOI:** 10.3390/v10060318

**Published:** 2018-06-11

**Authors:** Dániel Baráth, Nikoletta Jaksa-Czotter, János Molnár, Tünde Varga, Júlia Balássy, Luca Krisztina Szabó, Zoltán Kirilla, Gábor E. Tusnády, Éva Preininger, Éva Várallyay

**Affiliations:** 1 Agricultural Biotechnology Institute, NARIC, 2100 Gödöllő, Hungary; barath.daniel@abc.naik.hu (D.B.); czotter.nikoletta@abc.naik.hu (N.J.-C.); varga.tunde07@gmail.com (T.V.); sargarigofutty@gmail.com (J.B.); 2 Department of Biotechnology, Nanophagetherapy Center, Enviroinvest Corporation, 7632 Pécs, Hungary; molnarjanesz@gmail.com; 3 Fruitculture Research Institute, NARIC, 1223 Budapest, Hungary; szabo.luca.krisztina@fruitresearch.naik.hu (L.K.S.), zoltan.kirilla@gmail.com (Z.K.); e.preininger@resinfru.hu (É.P.); 4 Institute of Enzymology, Research Center of Natural Sciences, HAS, 1117 Budapest, Hungary; tusnady.gabor@ttk.mta.hu

**Keywords:** small RNA NGS, metagenomics, diagnostics, fruit tree viruses, Cherry virus A, little cherry virus 1, apricot

## Abstract

Fruit trees, such as apricot trees, are constantly exposed to the attack of viruses. As they are propagated in a vegetative way, this risk is present not only in the field, where they remain for decades, but also during their propagation. Metagenomic diagnostic methods, based on next generation sequencing (NGS), offer unique possibilities to reveal all the present pathogens in the investigated sample. Using NGS of small RNAs, a special field of these techniques, we tested leaf samples of different varieties of apricot originating from an isolator house or open field stock nursery. As a result, we identified Cherry virus A (CVA) and little cherry virus 1 (LChV-1) for the first time in Hungary. The NGS results were validated by RT-PCR and also by Northern blot in the case of CVA. Cloned and Sanger sequenced viral-specific PCR products enabled us to investigate their phylogenetic relationships. However, since these pathogens have not been described in our country before, their role in symptom development and modification during co-infection with other viruses requires further investigation.

## 1. Introduction

Apricot (*Prunus armeniaca*) is one of the most popular fruits in central Europe, especially in Hungary, where it is not only consumed as a fresh fruit, but also serves as a raw material for jam and “Palinka” (a distilled spirit) production. Thanks to intensive breeding programs since 1950, many varieties have become available with improved characteristics for these specific purposes. In accordance with the usual routine [1], mother trees of the new, approved varieties free from viruses are kept in isolator houses to prevent subsequent exposure to viruses, especially the Plum pox virus (PPV), and infections. They are also kept in open field stock nurseries which provide propagation material for the future.

Apricot trees can be infected by different viral pathogens, among them PPV, the Prunus necrotic ringspot virus, Prune dwarf virus, Apricot latent virus, Apple mosaic virus and Apple chlorotic leaf spot virus. These are all regulated viruses in Hungary, whose presence must be checked regularly in commercialized nurseries [2]. For these tests, traditional virus diagnostic methods (DAS-ELISA and RT-PCR) are used which are sensitive, but have critical limitations, as they are only able to detect infection of the tested pathogens [1]. In the last decade, sequencing techniques have rapidly developed, including next generation sequencing (NGS) based diagnostics of fruit tree viruses [3,4,5,6]. NGS makes it possible to sequence all of the genetic material present in the sample (belonging to both the host and all infecting pathogens) [7]. This information can be used not only for virus diagnostics, but also for metagenomics studies [7,8]. NGS analyses of symptomatic trees led to the identification of new variants or completely new *Prunus* infecting viruses [9,10,11,12,13] and disclosed the simultaneous presence of different variants and their recombination origin [10,14,15]. With the help of NGS, virus presence without visible symptoms could be identified from new locations [16] and new hosts [17,18]. Small RNA (sRNA) NGS is a special field of metagenomics, based on sequencing of sRNA products derived from RNA-based defense reaction (RNA interference (RNAi)) of the host plant. In a virus-infected plant, RNAi produces small interfering (si) RNAs (21–24 nt long) with the same sequence information as the infecting viruses [19,20]. Sequence analysis of the siRNA population of the host plant can disclose the presence of any viral pathogen with high sensitivity [19,21]. As a diagnostic tool, sRNA seq was proved to have the same sensitivity as ribodepleted RNAseq [22] and was found to be 10× more sensitive than RT-PCR [23]. Analysis of the sRNA population in samples extracted directly from field plants offers a unique opportunity to identify viroids [24] or viruses [25] even if they are alien to the plant or never described [26,27], facilitating the efficient survey of plantations [28]. 

Cherry virus A (CVA) was first described from sweet cherry in Germany by Jelkman [29]. Since that time, this capillovirus (genus in the *Betaflexivirideae* family) has been reported all over the world and was found to be frequent on the sweet and sour cherry [30,31]. Moreover, it was detected in several *Prunus* hosts (apricot, peach, plum, Japanese apricot) as well [31,32,33]. Its detection is not possible either by biological indexing or by ELISA. With the appearance of NGS, it is often reported to be present in mixed infections, together with other well-known fruit tree-infecting viruses, making it difficult to correlate its presence with the occurring symptoms [30,31]. Although it is considered to be latent, it cannot be ruled out that the severity of symptoms can be affected by its presence. Until now, it has no identified vectors, but is readily transmitted by grafting, thus its presence should be avoided in stock nurseries [30,31]. CVA has a positive RNA genome that includes two ORFs. ORF1 encodes the replicase and the coat protein, while ORF2 in a different frame contains a motif for a (putative) movement protein [31].

Little cherry virus 1 (LChV-1), a member of the *Velarivirus* genus in the *Closterovirideae* family, was first reported from cherries showing little cherry disease [34]. Since that time, the presence of its distinct variants has been connected with other disorders such as Kwanzan stunting [35] and Shirofugen stunt disease [36]. Infection of cherry by LChV-1 has been reported all over the world [37,38,39,40], usually as a coinfection with CVA. Moreover, its presence was also reported from different hosts (almond, peach and plum) [35,41] and recently from apricot [17]. It is graft-transmittable and although its close relative LChV-2 is transmitted by mealybugs, LChV-1 still lacks a confirmed vector. Its positive sense RNA genome has eight ORFs, including ORF3 encoding a heat-shock protein 70 homologue (HSP70h) and ORF5 and ORF6 encoding two coat proteins [41].

During our work, we used sRNA NGS to survey virus infections in apricot stock nurseries and have found and validated the presence of CVA and LChV-1, which have never before been detected in Hungary.

## 2. Materials and Methods

### 2.1. Plant Material, Sample Preparation

Samples were collected from an isolator house and open field stock nursery in Érd, at the Research Station of the Fruticulture Research Institute of NARIC. Leaf samples from four different branches of the tree, from three different varieties—Ligeti óriás (Parkland giant), Pannónia kajszi (Pannonian apricot), Magyar kajszi (Hungarian apricot)—were collected. Leaf samples of the *in vitro* cultured plantlets from all three varieties were also collected. RNA was extracted from leaf samples by the CTAB method [42]. RNA pools, representing each variety at different locations (isolator house or stock nursery) were prepared by mixing equal amounts of RNA originating from different individuals. These pools were used for sRNA library preparation (five libraries in total) using Truseq Small RNA Library Preparation Kit (Illumina, San Diego, CA, USA) and our modified protocol [42]. Samples were sequenced using a single index on a HiScan2000 by UD Genomed (Debrecen, Hungary) 50 bp, single end (8 samples/1 sequencing lane). Fastq files of the sequenced libraries were deposited to the GEO and can be accessed through series accession number GSE114251.

### 2.2. Pipeline for Data Evaluation of NGS Results (Bioinformatics)

For bioinformatics analysis, we used our published pipeline [42]. Briefly, the resulting reads were sorted according to their indexes. Adapters of the sequenced reads were removed by the Trimmomatic program [43], their quality was checked by the FastQC program (http://www.bioinformatics.babraham.ac.uk/projects/fastqc) and deduplicated by the Picard MarkDuplicates tool (http://broadinstitute.github.io/picard). For virus detection, we used two different pipelines in parallel: (A) short reads were mapped to viral reference genomes (Refseq viral database of NCBI from only plant and invertebrate hosts were used) by the BWA-aln short read aligner [44] with default options. Mapped reads were counted both with and without deduplication using samtools idxstats [45]. Redundant reads of the resultant hits were normalized to read/million read. Consensus viral sequences from the aligned deduplicated reads were generated using the samtools/bcftools [45] pipeline. Coverage of the appropriate genome was counted as the percentage of the genome covered by nucleotide information from the mapped sRNA reads. (B) De novo assembling of the deduplicated reads was performed by using Velvet with k-mer: 13, 15, 17 [46]. The generated contigs were annotated by MegaBLAST [47] to the RefSeqs and nr (viral database of NCBI from plant and invertebrate hosts) of NCBI.

### 2.3. Validation of Predicted Virus Diagnostics by RT-PCR

cDNA was synthetized from pooled RNA extracts representing each library or each individual tree using a random primer of the RevertAid First Strand cDNA Synthesis Kit (Thermo Fisher Scientific, Waltham, MA, USA), according to the manufacturer’s instructions. The generated cDNA was used as template for PCR reactions using Phire Hot Start II DNA Polymerase (Thermo Fisher Scientific) and published diagnostic primer (for PPV, [48]) or new ones (see Table S1) which were designed according to the sequenced sRNA reads. PCR products were analysed by agarose gel electrophoresis. For Sanger sequencing, cDNA was synthetized from pooled RNA extracts of individual plants and virus-specific PCR was done using Phusion Hot Start II High-Fidelity DNA Polymerase (Thermo Fisher Scientific). The purified products were cloned into pGEM^®^-T Vector System I (Promega, Fitchburg, WI, USA) and sequenced. Sequences were deposited into GenBank (GenBank Accession Numbers: MH321189-91.).

### 2.4. Phylogenetic Analysis

To compare sequenced and cloned PCR products, we used MEGA 7.0.21 [49] with the implemented neighbor-joining algorithm. The percentage of replicate trees in which the associated taxa clustered together in the bootstrap test (500 replicates) are shown next to the branches.

### 2.5. Validation by Northern Blot

For Northern blot analyses, 3 µg of total RNA was separated on formaldehyde-1.5% agarose gel and blotted to Amersham Hybond-NX membrane (GE Healthcare, Chicago, IL, USA), by the capillary method using 20× SSC (3 M NaCl and 0.3 M Na-citrate; pH 7.0). Hybridization was carried out at 65 °C in Church buffer (0.5 M Sodium Phosphate buffer, pH 7.2 containing 1% BSA, 1 mM EDTA, 7% SDS) overnight with the appropriate radioactively labelled probe, washed for 5 min in 2× SSC, 0.1% SDS and for 15 min in 0.5× SSC, 0.1% SDS at the temperature of the hybridization and exposed to an X-ray film. Virus-specific, P32-labelled DNA probes were prepared by using the DecaLabel DNA Labelling Kit (Thermo Fischer Scientific). As a template for Northern blot, probe PCR-amplified and purified product of the cloned region of CVA was used.

## 3. Results and Discussion

### 3.1. Small RNA NGS Revealed the Presence of CVA and LChV-1 

#### 3.1.1. Initial Statistics

Our samples originated from a stock nursery hosted by the Fruit Research Institute of NARIC. We sampled three apricot varieties, bred in Hungary: Ligeti óriás (LO–Parkland giant), Pannónia kajszi (P–Pannonian apricot) and Magyar kajszi (M–Hungarian apricot). Individuals, both under the isolator net and on open field, were sampled (Table S2). *In vitro* cultures which served as a propagation material for the mother trees were also available and tested. RNA pools were prepared from the RNA extracts of leaves according to the plant varieties and places of origin. In total, five sRNA libraries were sequenced by Illumina HiScan (Magyar kajszi variety from the isolator was not sequenced). As a result, 11.9–13.3 million raw reads/library were generated (Table S3). After quality control and trimming of the adapters, duplicates were removed and non-redundant reads (530,000–1.7 million/library), without removing the host-specific sRNAs, were used for virus diagnostics. In different libraries, 4.75–22.86% of the total non-redundant reads were mapped to viral reference genomes. In parallel, in the five libraries, 3444–16,284 contigs were assembled with different k-mers (13, 15, 17) using Velvet (Table S4), resulting in only 0–154 contigs of viral origin.

#### 3.1.2. Small RNA NGS-Based Virus Diagnostics

In order to identify viruses in the samples, sRNA reads or longer contigs, were aligned and mapped to reference genomes and partial sequences of viruses originating from plant or insect hosts. During this analysis, among all the contigs, PPV-, CVA- and LChV-1-specific contigs were identified (Table S4). The length of the contigs ranged from 31–188 nt. In 2_LO_sn, there were 14 and in 5_M_sn 128 PPV-specific contigs, there were only six and eight CVA- and 26 LChV-1-specific contigs in 3_P_ih, 4_P_sn and 5_M_sn, respectively (Table S5). When sRNA reads were directly aligned to the viral reference genome, the number of matched non-redundant and normalized redundant reads was counted and coverage (in %) of the whole viral reference genome was also calculated (Table S5). According to our analysis, two libraries: 2_LO_sn and 5_M_sn represented plant materials that were infected with PPV. Besides PPV, we identified infection of CVA in 3_P_ih, 4_P_sn and LChV-1 in 5_M_sn libraries. Virus-specific reads originated from the entire genome in each case (Figure S1a,b upper panels). CVA- and LChV-1-specific reads were mainly 21 nt long with both sense and antisense origins, while more than one-third of the LChV-1-specific sRNA reads were 22 nt long, suggesting a key role of antiviral DCL4 and DCL2 during their biogenesis (Figure S1a,b lower panel) [20,25].

### 3.2. Validation of the Small RNA NGS Virus Diagnostics

In order to validate our deep sequencing results, we synthesized cDNA from pooled RNAs representing the sequenced libraries and set up RT-PCR reactions with published diagnostic primers (for PPV [48]) or with primers designed according to the sequenced sRNA reads (Table S1). Positive controls (cDNA from virus-containing samples) and negative controls were always included. According to sRNA NGS analyses, two libraries: Ligeti óriás and Pannónia kajszi, which originated from an open field stock nursery, were infected with PPV. To reveal how widespread this infection is, the presence of the virus was validated by RT-PCR using cDNA produced from the individual trees (Figure S2). According to this analysis, only one tree from each variety was infected. The presence of PPV was rare; it was missing from the isolator and occurred only at the open field; consequently, it could be the result of an onsite infection that originated from the neighborhood.

sRNA NGS also showed the presence of two additional viruses, never before described in Hungary: CVA and LChV-1.

#### 3.2.1. Validation of the Presence of Cherry Virus A

**Figure 1:**
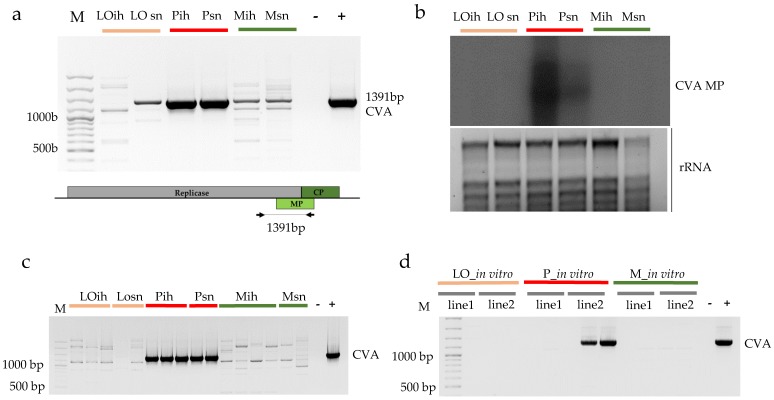
Validation of the presence of CVA in the samples. (**a**) RT-PCR (**b**) Northern blot of the pooled RNAs representing the sequenced libraries: LO-Ligeti óriás, P-Pannónia kajszi, M-Magyar kajszi, ih-isolator house, sn-stock nursery. (**a**) The lower panel represents the genome of the virus indicating the PCR amplified part; (**b**) The upper panel shows film exposed to the membrane hybridized with a CVA-specific radioactively labelled probe; the lower panel is a photo of the EtBr-stained 1.5% agarose gel from which the blot was prepared; rRNAs served as a loading control; (**c**) CVA-specific RT-PCR of the sampled individuals from which the sRNA libraries were produced; (**d**) CVA-specific RT-PCR of two batches of two different lines of the *in vitro* plantlets served as a progenitor of the production of the mother trees under the isolator M- 100 bp Plus GeneRuler, (−) negative, (+) positive control.

The presence of CVA indicated by sRNA reads in 3_P_ih and 4_P_sn libraries could be validated by both RT-PCR, amplifying the entire putative MP region (Figure 1a) and Northern blot analysis (Figure 1b), using pooled RNA representing the sequenced libraries, indicating that Pannónia kajszi trees, even in the isolator house, were infected with this virus.

Moreover, each tested individual tree proved to be positive for CVA presence (Figure 1c). This result raised the possibility that this infection did not derive from the environment, but instead originated from the established *in vitro* cultures. Testing two batches of plantlets from two different lines of *in vitro* cultures of Pannónia kajszi variety line 2 showed the presence of the virus (Figure 1d), supporting this theory. As CVA is not on the quarantine list, its presence was not checked during sanitation. Even though its presence is not connected to any visual symptom, especially not on the apricot, it may interfere with other viruses; therefore, its presence should be avoided in any propagation materials.

#### 3.2.2. Validation of the Presence of Little Cherry Virus 1

**Figure 2:**
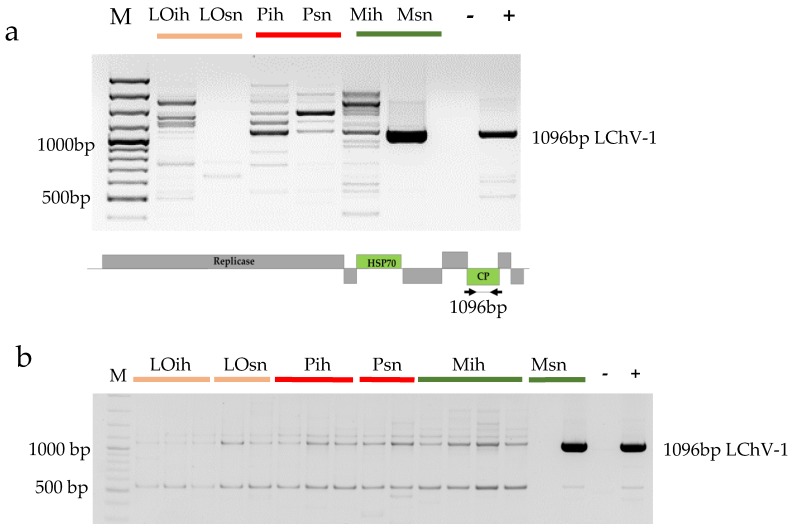
RT-PCR validation of the presence of LChV-1 in the (**a**) pooled individual RNAs representing the sequenced libraries and (**b**) RNAs from individual trees: LO-Ligeti óriás, P-Pannónia kajszi, M-Magyar kajszi, ih-isolator house, sn-stock nursery. (**a**) The lower panel represents the genome of the virus indicating the PCR-amplified part, M- 100 bp Plus Gene Ruler, (−) negative, (+) positive control.

Apricot was not considered as a natural host of LChV-1; moreover, the mechanical inoculation of this host with this virus failed [50], but recently its presence on apricots was reported in the Czech Republic [17]. With sRNA NGS, we detected its presence in the 5_M_sn library, produced from trees of the Magyar kajszi variety on the open field stock nursery. The presence of the virus was validated by RT-PCR (Figure 2a). 

Although Magyar kajszi trees in the isolator were not NGS sequenced, according to RT-PCR they were found to be uninfected with LChV-1 (Figure 2a). Moreover, RT-PCR of the individuals showed that only one tree in an open field was infected (Figure 2b). To find out whether *in vitro* clones of this variety contain the virus, we checked two batches of two different lines of the *in vitro* cultures for the presence of LChV-1 and we did not find any infection. These findings suggest that infection of the apricot occurred in the open field stock nursery and not by grafting during its propagation. Although its vector transmission is not proven, LChV-1 could be transmitted by insect vectors [51]. The apricot stock nursery is close to a sweet and sour cherry variety collection, which could serve as a reservoir for the virus. However, the infection rate was low and we found LChV-1 in Magyar kajszi, one of the varieties which was shown to be infected in the Czech Republic. This coincidence, even in the lack of symptoms, suggests the susceptibility of this variety to LChV-1.

Results of the sRNA NGS and its comparison with RT-PCR, summarized in Table 1, show that this high-throughput method can be reliably used for virus diagnostics. 

#### 3.2.3. Phylogenetic Relationship of Hungarian CVA and LChV-1 Isolates

During RT-PCR validation, cDNAs were not only synthesized from RNA pools, but from RNAs extracted from individuals as well. Using these individual specific cDNAs, the PCR experiment was repeated by using a proofreading DNA polymerase. Amplified products were cloned and sequenced by traditional Sanger sequencing. Sequences were deposited into GenBank (Accession numbers: MH321189-91) and used for phylogenetic comparison (Table 2). 

**Figure 3:**
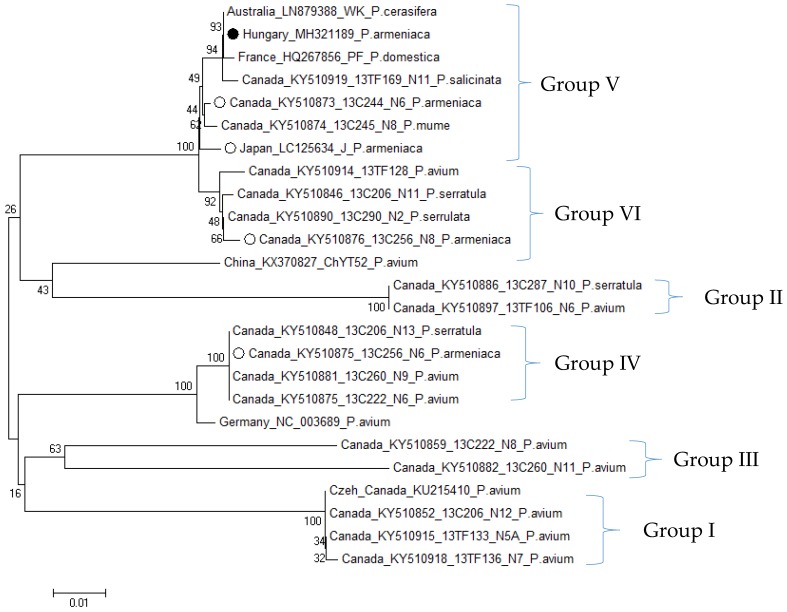
Phylogenetic analysis of movement protein coding sequences of CVA. ●-Sequences from Hungarian apricot, Ο-Sequences from *P. armeniaca* origin. Bootstrap values are indicated as percentages.

According to the phylogenetic analysis of the MP coding region of the CVA isolate from Pannónia kajszi, this isolate belongs to Group V, together with isolates from non-cherry hosts and more importantly together with other CVA isolates originating from the *P. armeniaca* host (Figure 3). Pairwise comparison of CVA sequences from this host showed high similarity (98–99% nt identity), except for one Canadian isolate (16C256_N6), which has a higher diversity (92% identity for this part of the movement protein coding region), supporting the observation of Gao which questioned the origin of the mode of exchange between and the adaptation of CVA to various hosts (Table S6) [30].

**Figure 4:**
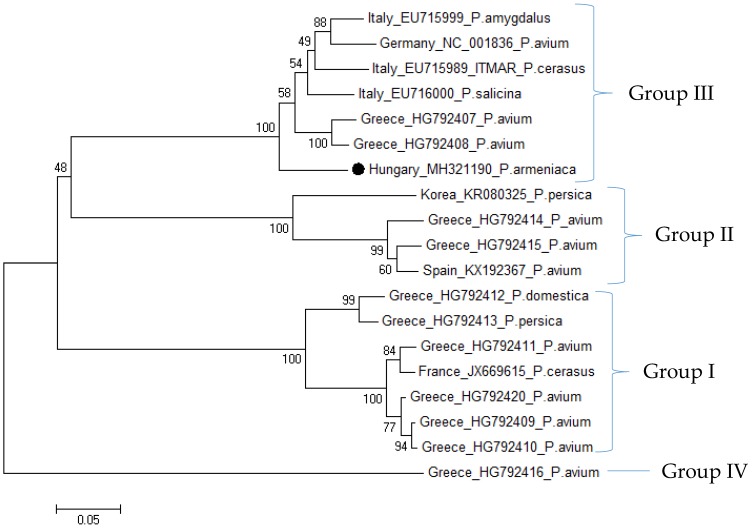
Phylogenetic analysis of the CP coding region of LChV-1 sequences. ●-Sequence from Hungarian apricot. Bootstrap values are indicated as percentages.

Phylogenetic analysis of Hungarian LChV-1 isolate (both according to HSP70h and CP sequences) shows that it is very closely related to the Italian isolate ITMAR, which has been found to be the main causative agent of Kwanzan stunt disease, clustering to Group III (Figure 4) [35]. As a result of a comparison of sequences derived from different countries and hosts, the LChV-1 partial CP coding region from the Hungarian apricot showed the highest similarity to the isolate EU716000 from Italy from *P. salicina* and HG792407 from Greece from *P. avium*. Comparative analysis of the HSP70h coding part of the genome showed that the German (NC_001836) and the Italian (EU715989) ITMAR isolates showed the highest similarity to the Hungarian HSP70h sequence (Table S7), supporting their close relation in Group III, as demonstrated by the phylogenetic analysis comparison of their CP coding sequences. Unfortunately, the Czech LChV-1 isolate from apricot has no available sequence data for HSP70h or the CP region, hindering the identification of its relationship to the Hungarian isolate.

## 5. Conclusions

New high-throughput sequencing-based methods can provide valuable information about the presence of different viral pathogens. Due to their speed and reliability, they proved to be a suitable alternative to labor- and time-intensive biological indexing [52,53]. In addition to dsRNA seq, here we show that other NGS-based techniques, such as sRNA NGS, can also be reliably used for virus diagnostics in woody plants, such as fruit trees. Results gained by sRNA NGS could be validated by other molecular biology methods such as RT-PCR and Northern blot. Although sensitivity of NGS can sometimes lead to false positive results [54], our results imply that virus elimination during the production of the propagation material is essential and needs to be monitored by the most sensitive virus diagnostic methods in order to minimize the possibility of viruses passing through this control. sRNA NGS is one alternative which can be used during the production of virus-free propagation material to avoid unknown and unwanted infections. However, before becoming a diagnostic tool, adopted in the certification protocols, apart from the reduction of the sequencing cost, standardization and improvement of the bioinformatics pipeline is highly needed as well [55]. 

sRNA NGS technology helped us to describe the unknown and rare presence of LChV-1, in apricot. The virus diagnostics of the *in vitro* cultured plant, trees under the isolator net and in an open field in the stock nurseries suggested that while infection with LChV-1 happened in an open field from the surroundings, CVA infection originated from the propagation material. However, to test this hypothesis, the presence of LChV-1 in the surrounding cherry and sour cherry plantations together with the presence of mealybugs or other possible vectors should be tested.

In this work, CVA and LChV-1 were first described in Hungary. To get a more detailed picture of their prevalence and distribution in different cultivated *Prunus* species and species present in the natural vegetation, further investigation is necessary.

## Figures and Tables

**Table 1:** Summary of the small RNA NGS virus diagnostics and its RT-PCR validation.

Variety	Type of Analysis	Origin of Samples		
		*In Vitro*	Isolator House	Stock Nursery
Ligeti óriás	NGS virus hit	-	0	PPV
	PCR *	0	0	1/2 PPV
Pannónia	NGS virus hit	-	CVA	CVA
	PCR *	2/4 CVA	3/3 CVA	2/2 CVA
Magyar kajszi	NGS virus hit	-	0	PPV, LChV-1
	PCR *	0	0	1/2 PPV, 1/2 LChV-1

0—no hit; * number of positive/tested individuals.

**Table 2:** Sequences of the amplified part of viruses uploaded into GenBank with their identifier.

Virus	Variety	Genebank Identifier	Genome Used as a Reference	Position on the Reference Genome	Function of the Amplified Part of the Genome	Identity on Nucleotide Level (%)	Identity on Amino Acid Level (%)
CVA	Pannónia kajszi/Pannonian apricot	MH321189	NC_003689.1	5401–6791	replicase (partial)	1275/1392(92%)	392/463(85%)
				5400–6791	movement protein		421/463(91%)
LChV-1	Magyar kajszi/Hungarian apricot	MH321190	NC_001836.1	12,165–13,261	coat protein	1011/1097(92%)	338/365(93%)
LChV-1	Magyar kajszi/Hungarian apricot	MH321191	NC_001836.1	9493–10,288	HSP70h	753/796(95%)	251/265(95%)

## Supplementary Materials

The following are available online at http://www.mdpi.com/1999-4915/10/6/318/s1. Figure S1: Schematic representation of the (a) CVA and (b) LChV-1 specific sRNA reads, Figure S2: RT-PCR validation of the presence of PPV in the sampled individual trees using primers amplifying 242 bp of the coat protein; Table S1: Sequence of the used of PCR primers for virus detection with their appropriate references, Table S2: Basic information of the sampled varieties with the number of the small RNA library, Table S3: Initial statistics of the sequenced reads of different libraries, Table S4: Initial statistics of the velvet built contigs, Table S5: Summary of bioinformatics analysis, Table S6: Percent identity matrix from pairwise comparison of the sequences available at GenBank coding MP of the CVA, originating from *P. armeniaca* host, Table S7: Percent identity matrix from pairwise comparison of the sequences available at GenBank coding HSP70 of the LChV-1.

## References

[1] Varveri C., Maliogka V.I., Kapari-Isaia T., Loebenstein G., Katis N.I. (2015). Chapter One—Principles for Supplying Virus-Tested Material. Advances in Virus Research.

[2] Barba M., Ilardi V., Pasquini G., Loebenstein G., Katis N.I. (2015). Chapter Three—Control of Pome and Stone Fruit Virus Diseases. Advances in Virus Research.

[3] Barba M., Czosnek H., Hadidi A. (2014). Historical Perspective, Development and Applications of Next-Generation Sequencing in Plant Virology. Viruses.

[4] Boonham N., Kreuze J., Winter S., van der Vlugt R., Bergervoet J., Tomlinson J., Mumford R. (2014). Methods in virus diagnostics: From ELISA to next generation sequencing. Virus Res..

[5] Hadidi A., Flores R., Candresse T., Barba M. (2016). Next-Generation Sequencing and Genome Editing in Plant Virology. Front. Microbiol..

[6] Jones S., Baizan-Edge A., MacFarlane S., Torrance L. (2017). Viral Diagnostics in Plants Using Next Generation Sequencing: Computational Analysis in Practice. Front. Plant Sci..

[7] Roossinck M.J. (2017). Deep sequencing for discovery and evolutionary analysis of plant viruses. Virus Res..

[8] Roossinck M.J., Martin D.P., Roumagnac P. (2015). Plant Virus Metagenomics: Advances in Virus Discovery. Phytopathology.

[9] Elbeaino T., Giampetruzzi A., De Stradis A., Digiaro M. (2014). Deep-sequencing analysis of an apricot tree with vein clearing symptoms reveals the presence of a novel betaflexivirus. Virus Res..

[10] Marais A., Faure C., Candresse T. (2016). New Insights into Asian Prunus Viruses in the Light of NGS-Based Full Genome Sequencing. PLoS ONE.

[11] Marais A., Faure C., Mustafayev E., Barone M., Alioto D., Candresse T. (2014). Characterization by Deep Sequencing of Prunus virus T, a Novel Tepovirus Infecting Prunus Species. Phytopathology.

[12] Villamor D.E.V., Pillai S.S., Eastwell K.C. (2017). High throughput sequencing reveals a novel fabavirus infecting sweet cherry. Arch. Virol..

[13] He Y., Cai L., Zhou L., Yang Z., Hong N., Wang G., Li S., Xu W. (2017). Deep sequencing reveals the first fabavirus infecting peach. Sci. Rep..

[14] Marais A., Faure C., Mustafayev E., Candresse T. (2015). Characterization of New Isolates of Apricot vein clearing-associated virus and of a New Prunus-Infecting Virus: Evidence for Recombination as a Driving Force in Betaflexiviridae Evolution. PLoS ONE.

[15] Koloniuk I., Sarkisova T., Petrzik K., Lenz O., Přibylová J., Fránová J., Špak J., Lotos L., Beta C., Katsiani A. (2018). Variability Studies of Two Prunus-Infecting Fabaviruses with the Aid of High-Throughput Sequencing. Viruses.

[16] Šafářová D., Faure C., Marais A., Suchá J., Paprštein F., Navrátil M., Candresse T. (2017). First Report of Prunus virus F Infecting Sour Cherry in the Czech Republic. Plant Dis..

[17] Šafářová D., Faure C., Candresse T., Navrátil M., Nečas T., Marais A. (2016). First Report of Little cherry virus 1 Infecting Apricot in the Czech Republic. Plant Dis..

[18] James D., Phelan J., Jesperson G. (2018). First Report of Prunus virus F infecting sweet cherry (*Prunus avium* cv. ‘StaccatoTM’) in Canada. Plant Dis..

[19] Donaire L., Wang Y., Gonzalez-Ibeas D., Mayer K.F., Aranda M.A., Llave C. (2009). Deep-sequencing of plant viral small RNAs reveals effective and widespread targeting of viral genomes. Virology.

[20] Parent J.-S., Martinez de Alba A.E., Vaucheret H. (2012). The origin and effect of small RNA signaling in plants. Front. Plant Sci..

[21] Kreuze J.F., Perez A., Untiveros M., Quispe D., Fuentes S., Barker I., Simon R. (2009). Complete viral genome sequence and discovery of novel viruses by deep sequencing of small RNAs: A generic method for diagnosis, discovery and sequencing of viruses. Virology.

[22] Pecman A., Kutnjak D., Gutierrez-Aguirre I., Adams I., Fox A., Boonham N., Ravnikar M. (2017). Next Generation Sequencing for Detection and Discovery of Plant Viruses and Viroids: Comparison of Two Approaches. Front. Microbiol..

[23] Santala J., Valkonen J.P.T. (2018). Sensitivity of Small RNA-Based Detection of Plant Viruses. Front. Microbiol..

[24] Navarro B., Pantaleo V., Gisel A., Moxon S., Dalmay T., Bisztray G., Di Serio F., Burgyan J. (2009). Deep sequencing of viroid-derived small RNAs from grapevine provides new insights on the role of RNA silencing in plant-viroid interaction. PLoS ONE.

[25] Pantaleo V., Saldarelli P., Miozzi L., Giampetruzzi A., Gisel A., Moxon S., Dalmay T., Bisztray G., Burgyan J. (2010). Deep sequencing analysis of viral short RNAs from an infected Pinot Noir grapevine. Virology.

[26] Giampetruzzi A., Roumi V., Roberto R., Malossini U., Yoshikawa N., La Notte P., Terlizzi F., Credi R., Saldarelli P. (2012). A new grapevine virus discovered by deep sequencing of virus- and viroid-derived small RNAs in Cv Pinot gris. Virus Res..

[27] Wu Q., Wang Y., Cao M., Pantaleo V., Burgyan J., Li W.X., Ding S.W. (2012). Homology-independent discovery of replicating pathogenic circular RNAs by deep sequencing and a new computational algorithm. Proc. Natl. Acad. Sci. USA.

[28] Czotter N., Molnar J., Szabó E., Demian E., Kontra L., Baksa I., Szittya G., Kocsis L., Deak T., Bisztray G. (2018). NGS of Virus-Derived Small RNAs as a Diagnostic Method Used to Determine Viromes of Hungarian Vineyards. Front. Microbiol..

[29] Jelkmann W. (1995). Cherry virus A: CDNA cloning of dsRNA, nucleotide sequence analysis and serology reveal a new plant capillovirus in sweet cherry. J. Gen. Virol..

[30] Gao R., Xu Y., Candresse T., He Z., Li S., Ma Y., Lu M. (2017). Further insight into genetic variation and haplotype diversity of Cherry virus A from China. PLoS ONE.

[31] Marais A., Svanella D.L., Barone M., Gentit P., Faure C., Charlot G., Ragozzino A., Candresse T. (2012). Development of a polyvalent RT-PCR detection assay covering the genetic diversity of *Cherry capillovirus* A. Plant Pathol..

[32] Kesanakurti P., Belton M., Saeed H., Rast H., Boyes I., Rott M. (2017). Comparative analysis of cherry virus A genome sequences assembled from deep sequencing data. Arch. Virol..

[33] Marais A., Faure C., Svanella-Dumas L., Candresse T. (2008). First Report of Cherry virus A in *Prunus mume* in China. Plant Dis..

[34] Keim-Konrad R., Jelkmann W. (1996). Genome analysis of the 3′-terminal part of the little cherry disease associated dsRNA reveals a monopartite clostero-like virus. Arch. Virol..

[35] Matic S., Minafra A., Sánchez-Navarro J.A., Pallás V., Myrta A., Martelli G.P. (2009). ‘Kwanzan Stunting’ syndrome: Detection and molecular characterization of an Italian isolate of Little cherry virus 1. Virus Res..

[36] Candresse T., Marais A., Faure C., Gentit P. (2013). Association of Little cherry virus 1 (LChV1) with the Shirofugen Stunt Disease and Characterization of the Genome of a Divergent LChV1 Isolate. Phytopathology.

[37] Glasa M. (2015). First report of little cherry virus-1 in Slovakia. J. Plant Pathol..

[38] Sabanadzovic S., Aboughanem N., Rowhani A., Grant J.A., Uyemoto J. (2005). Detection of Cherry virus A, Cherry necrotic rusty mottle virus and Little cherry virus 1 in California orchards. J. Plant Pathol..

[39] Komorowska B., Cieślińska M. (2004). First Report of Cherry virus A and Little cherry virus-1 in Poland. Plant Dis..

[40] Bajet N.B., Unruh T.R., Druffel K.L., Eastwell K.C. (2008). Occurrence of Two Little Cherry Viruses in Sweet Cherry in Washington State. Plant Dis..

[41] Katsiani A.T., Maliogka V.I., Amoutzias G.D., Efthimiou K.E., Katis N.I. (2015). Insights into the genetic diversity and evolution of Little cherry virus 1. Plant Pathol..

[42] Czotter N., Molnár J., Pesti R., Demián E., Baráth D., Varga T., Várallyay É., Pantaleo V., Chiumenti M. (2018). Use of siRNAs for Diagnosis of Viruses Associated to Woody Plants in Nurseries and Stock Collections. Viral Metagenomics: Methods and Protocols.

[43] Bolger A.M., Lohse M., Usadel B. (2014). Trimmomatic: A flexible trimmer for Illumina sequence data. Bioinformatics.

[44] Li H., Durbin R. (2009). Fast and accurate short read alignment with Burrows–Wheeler transform. Bioinformatics.

[45] Li H. (2011). A statistical framework for SNP calling, mutation discovery, association mapping and population genetical parameter estimation from sequencing data. Bioinformatics.

[46] Zerbino D.R., Birney E. (2008). Velvet: Algorithms for de novo short read assembly using de Bruijn graphs. Genome Res..

[47] Morgulis A., Coulouris G., Raytselis Y., Madden T.L., Agarwala R., Schaffer A.A. (2008). Database indexing for production MegaBLAST searches. Bioinformatics.

[48] Wetzel T., Candresse T., Ravelonandro M., Dunez J. (1991). A polymerase chain reaction assay adapted to plum pox potyvirus detection. J. Virol. Methods.

[49] Tamura K., Stecher G., Peterson D., Filipski A., Kumar S. (2013). MEGA6: Molecular Evolutionary Genetics Analysis Version 6.0. Mol. Biol. Evol..

[50] Wilks J.M., Welsh M.F. (1961). Host range studies of the little cherry disease virus. Can. J. Plant Sci..

[51] Jelkmann W., Fechtner B., Agranovsky A.A. (1997). Complete genome structure and phylogenetic analysis of little cherry virus, a mealybug-transmissible closterovirus. J. Gen. Virol..

[52] Rott M., Xiang Y., Boyes I., Belton M., Saeed H., Kesanakurti P., Hayes S., Lawrence T., Birch C., Bhagwat B. (2017). Application of Next Generation Sequencing for Diagnostic Testing of Tree Fruit Viruses and Viroids. Plant Dis..

[53] Al Rwahnih M., Daubert S., Golino D., Islas C., Rowhani A. (2015). Comparison of Next-Generation Sequencing Versus Biological Indexing for the Optimal Detection of Viral Pathogens in Grapevine. Phytopathology.

[54] Massart S., Candresse T., Gil J., Lacomme C., Predajna L., Ravnikar M., Reynard J.-S., Rumbou A., Saldarelli P., Škorić D. (2017). A Framework for the Evaluation of Biosecurity, Commercial, Regulatory, and Scientific Impacts of Plant Viruses and Viroids Identified by NGS Technologies. Front. Microbiol..

[55] Massart S., Olmos A., Jijakli H., Candresse T. (2014). Current impact and future directions of high throughput sequencing in plant virus diagnostics. Virus Res..

