# Supplementary material for: Small RNA NGS Revealed the Presence of Cherry Virus A and Little Cherry Virus 1 on Apricots in Hungary
Source: Viruses. 2018 Jun 11;10(6):318. doi: 10.3390/v10060318 (PMC6024520; doi:10.3390/v10060318)
Supplement: Supplementary file 1 [file viruses-10-00318-s001.zip › Figure S1-S2.docx]

Supplementary Figures to

Small RNA NGS revealed the presence of Cherry virus A and Little cherry virus 1 on apricot in Hungary

Dániel Baráth, Nikoletta Jaksa-Czotter, János Molnár, Tünde Varga, Júlia Balássy, Luca Krisztina Szabó, Zoltán Kirilla, Gábor E. Tusnády, Éva Preininger, Éva Várallyay*

*****Correspondence: varallyay.eva@abc.naik.hu

*
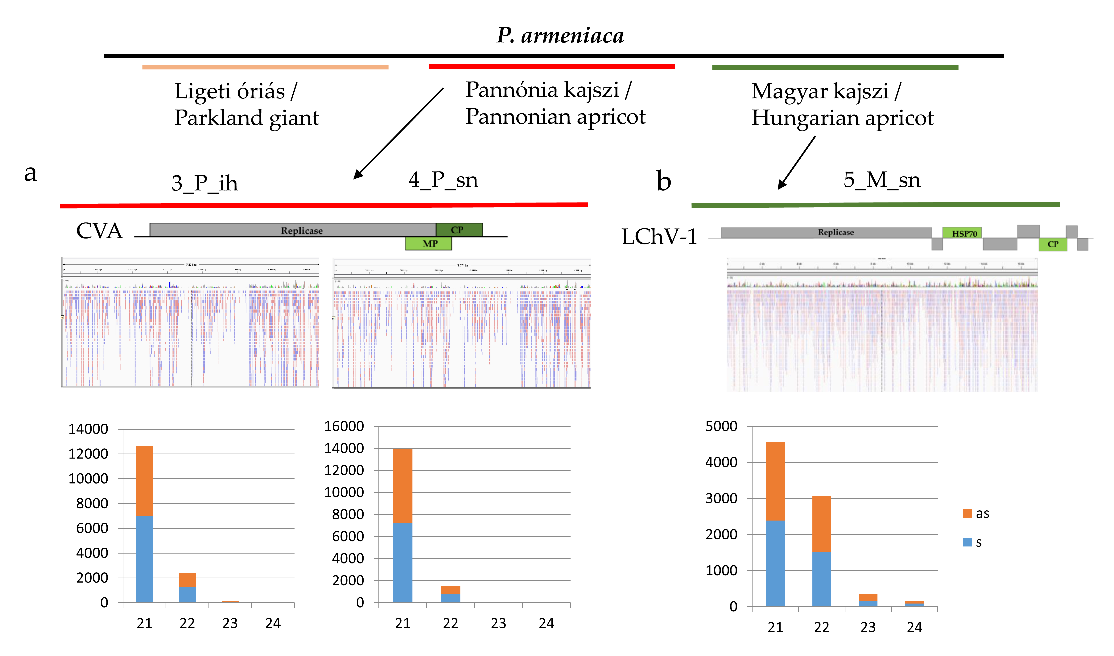
*

**Figure *S*1.** Schematic representation of the (a) CVA and (b) LChV-1 specific sRNA reads. Upper panels show the location of the sRNAs on the viral genome, lower panel shows the size distribution of the viral specific sRNA reads. Red –antisense, blue – sense orientation.

**
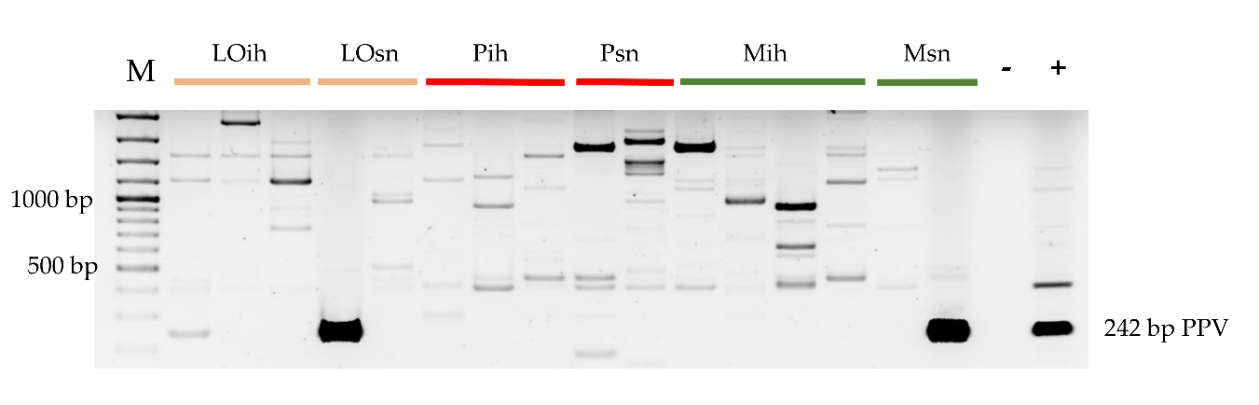
**

**Figure S2.** RT-PCR validation of the presence of PPV in the sampled individual trees using primers amplifying 242bp of the coat protein. LO-Ligeti óriás, P-Pannónia, M-Magyar, ih-isolator house, sn-stock nursery. M- 100bp+GeneRuler, (-) negative, (+) positive control.
